# QSAR and Docking Studies on Capsazepine Derivatives for Immunomodulatory and Anti-Inflammatory Activity

**DOI:** 10.1371/journal.pone.0100797

**Published:** 2014-07-08

**Authors:** Aparna Shukla, Pooja Sharma, Om Prakash, Monika Singh, Komal Kalani, Feroz Khan, Dnyaneshwar Umrao Bawankule, Suaib Luqman, Santosh Kumar Srivastava

**Affiliations:** 1 Metabolic and Structural Biology Department, CSIR-Central Institute of Medicinal and Aromatic Plants, P.O.- CIMAP, Kukrail Picnic Spot Road, Lucknow (Uttar Pradesh), India; 2 Molecular Bio-Prospection Department, CSIR-Central Institute of Medicinal and Aromatic Plants, P.O.- CIMAP, Kukrail Picnic Spot Road, Lucknow (Uttar Pradesh), India; 3 Analytical Chemistry Division, CSIR-Central Institute of Medicinal and Aromatic Plants, P.O.- CIMAP, Kukrail Picnic Spot Road, Lucknow (Uttar Pradesh), India; Wayne State University School of Medicine, United States of America

## Abstract

Capsazepine, an antagonist of capsaicin, is discovered by the structure and activity relationship. In previous studies it has been found that capsazepine has potency for immunomodulation and anti-inflammatory activity and emerging as a favourable target in quest for efficacious and safe anti-inflammatory drug. Thus, a 2D quantitative structural activity relationship (QSAR) model against target tumor necrosis factor-α (TNF-α) was developed using multiple linear regression method (MLR) with good internal prediction (r^2^ = 0.8779) and external prediction (r^2^_pred_ = 0.5865) using Discovery Studio v3.5 (Accelrys, USA). The predicted activity was further validated by *in vitro* experiment. Capsazepine was tested in lipopolysaccharide (LPS) induced inflammation in peritoneal mouse macrophages. Anti-inflammatory profile of capsazepine was assessed by its potency to inhibit the production of inflammatory mediator TNF-α. The *in vitro* experiment indicated that capsazepine is an efficient anti-inflammatory agent. Since, the developed QSAR model showed significant correlations between chemical structure and anti-inflammatory activity, it was successfully applied in the screening of forty-four virtual derivatives of capsazepine, which finally afforded six potent derivatives, CPZ-29, CPZ-30, CPZ-33, CPZ-34, CPZ-35 and CPZ-36. To gain more insights into the molecular mechanism of action of capsazepine and its derivatives, molecular docking and *in silico* absorption, distribution, metabolism, excretion and toxicity (ADMET) studies were performed. The results of QSAR, molecular docking, *in silico* ADMET screening and *in vitro* experimental studies provide guideline and mechanistic scope for the identification of more potent anti-inflammatory & immunomodulatory drug.

## Introduction

Capsicum species commonly known as chillies, relished as an important spice in the culinary art of the world and is known for its medicinal effect since the dawn of the human civilization. The medicinal property of ‘hot pepper’ has been attributed to the presence of capsaicin, a pungent principal ingredient produced as a secondary metabolite. Chemically known as 8-methyl-N-vanillyl-6-nonenamide. Capsaicin and its related compounds, collectively referred as ‘capsaicinoids’ or ‘vanilloids’, which bind specifically to transient receptor cation channel subfamily V (TRPV), that carry sensation of pain and responds naturally to noxious stimuli like high temperature and acidic pH [1]. Prolonged exposure causes nociceptor terminals to become insensitive to capsaicin, as well to other noxious stimuli [2]. Hyper stimulation of TRPV1 by capsaicin has an analgesic effect, since it leads to long-term desensitization of the sensory neurons. The clinical uses of TRPV1 agonist like capsaicin, are limited due to side effects of a burning sensation, irritation and neurotoxicity [3]. On the other hand, blocking of the pain-signalling pathway with a TRPV1 antagonist capsazepine represents a promising strategy for the development of novel analgesics with potentially fewer side effects [4]. Several non-neuronal effects of capsaicin have also been reported *viz*., induction of apoptosis in transformed cells [5], stimulation of prostaglandin formation leading to inhibition of gastric lesion [6], antibacterial activity [7], inhibition of cardiac excitability [8] and platelet aggregation [9]. Capsazepine is a known analog of capsaicin, discovered as a result of structure-activity relationship (SAR) studies [10]. Capsazepine induced similar action as capsaicin and resiniferatoxin (RTX) and exhibits even twofold more potent inhibition of expression of iNOS gene in LPS-stimulated murine macrophages through inactivation of NF-kB [11], [12]. NF-kB is a protein complex that control transcription of DNA and it is involved in cellular responses to stimuli such as stress, cytokines, free radicals, ultraviolet irradiations, oxidized low-density lipoprotein, and microbial antigens. NF-kB regulation of immune response and inflammation, cell lineage development, cell apoptosis, cell cycle progression and oncogenesis in response to stimuli have been shown to regulate the expression of several genes (bcl-2, bcl-xl), cellular inhibitor of apoptosis protein, tumor necrosis factor signalling pathway-related regulatory factor, cyclooxygenase-2 (COX-2), matrix metalloprotein peptide-9 (MMP-9) and inducible nitric oxide synthase (iNOS) and those for cell cycle regulatory components involved in tumorigenesis [13]–[18]. Therefore, in this work we have investigated the chemo preventive potential of capsazepine and its derivatives against pro-inflammatory mediator TNF-α through QSAR, *in vitro* activity evaluation and molecular docking studies, to understand the mechanism of action of vanilloids against inflammation and immunomodulation related to cancer. QSAR modelling also furnished the activity dependent structural descriptors and predicts the effective dose of other derivatives, thereby suggesting the possible toxicity range. Drugability of hit compounds was evaluated by using Lipinski's ‘Rule of Five’ and ADMET analysis through bioavailability filters.

## Materials and Method

### Dataset preparation

A total of 146 known TNF-α inhibitors were collected from various published literatures based on its structural diversity and activity coverage. The activity data for all compounds were taken from different scientific groups in terms of inhibitory activity (IC_50_ µM) [19]–[24]. 124 compounds out of 146, were selected as a training set based on following criteria to produce a good quantitative QSAR model: by covering a wide activity range of compounds and by including the most active, moderate and less active inhibitors (Table S1 in File S1). The biological activity for TNF-α inhibitors were ranging between 0.09 to 50 µM. The remaining 22 compounds were used as a test set to validate the generated model (Table S2 in File S1).

### Energy minimization

The structural drawing and geometry cleaning of the training set compounds were performed through, ChemBioOffice suite Ultra v12.0 (2010) software (CambridgeSoft Corp., UK). The compounds then subjected to energy minimization by using Discovery Studio v3.5 software (Accelrys Inc., USA) by applying CHARMm forcefield applicable to most of the small molecules. It adds several properties to the compounds including: initial potential energy, RMS gradient, CHARMm energy and minimization criteria.

### Chemical descriptors calculation

Molecular descriptors were calculated for each compounds using “Calculate Molecular Properties” module of the Discovery Studio v3.5 (Accelrys Inc., USA). These descriptors include 2D parameters (*e.g.*, AlogP, molecular weight, number of aromatic ring, number of H-acceptors, number of H-donors, number of rings, number of rotatable bonds, molecular fraction polar surface area) and 3D (Dipole and Jurs descriptor).

### Quantitative structure activity relationship (QSAR) model development

The set of energy optimised 146 compounds with calculated molecular properties were used for QSAR model development using create QSAR model module in Discovery Studio v3.5. Firstly all compounds were prepared for QSAR, and then the biological activities were specified as dependent property. Compounds were randomly divided into training (124 compounds) and test (22 compounds) set. This division was performed in such a manner that data coordinates of regression graph represent both training and test set compounds and distributed within the whole descriptor space of the entire dataset. Each data point of the test set showed closer match with the training set compounds. The regression model equation was derived by using statistical multiple linear regression approach (Table S3 in File S1).

### Model quality assessment and validation

**Figure 1:**
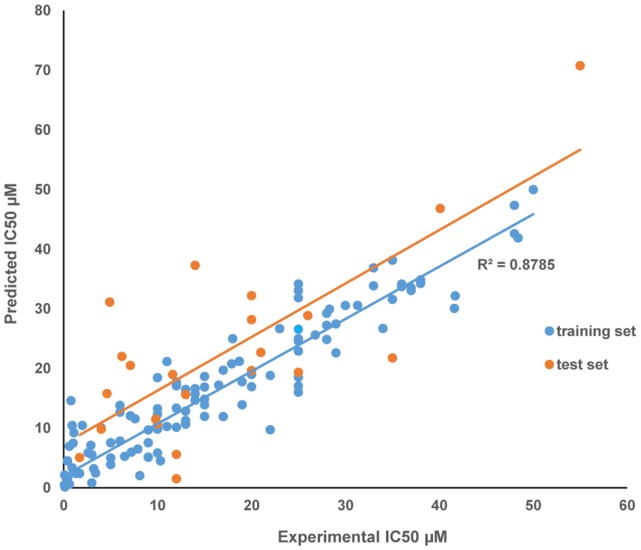
Graphical plot between experimental and predicted activities (IC_50_ µM) of the training and test set compounds. (A) Training data set (blue dots), (B) Test data set (orange dots).

The successful QSAR model must be robust enough to make accurate and reliable predictions of the non-investigated or query set compounds, therefore the obtained QSAR model from the training set should be subsequently validated. The conventional validation strategy for QSAR model analysis, based on multiple linear regression, include the calculation of cross validated squared correlation coefficient (r^2^) for internal validation and the predictive squared correlation coefficient (r^2^_pred_) for external validation. Here in this case the r^2^ was 0.878, and r^2^_pred_ was 0.5865, which ultimately prove the true predictability of model and the model was not obtained by chance only. The parameters for model construction were: (i) User set: AlogP, molecular weight, number of H-donors, number of H-acceptors, number of rotatable bonds, number of rings, number of aromatic rings, molecular fractional polar surface area, (ii) Initial model form: Least-Squares, (iii) Number of nearest neighbours: 20 and (iv) Dynamic smoothing factor: 0.5 Graphical plot between experimental and predicted activities (IC_50_ µM) of the training and test set compounds are represented in Figure 1.

#### QSAR analysis: Multiple linear regression equation

By considering the top eight descriptors following regression equation derived:
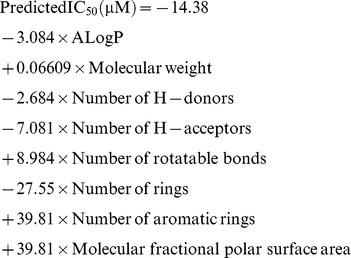





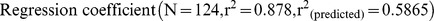



It is evident from the above mentioned equation that the molecular descriptors, ALogP, number of H-donors, number of H-acceptors and number of rings showed negative correlation with respect to the biological activity. On the other hand molecular weight, number of rotatable bonds, number of aromatic rings and molecular fractional polar surface area showed positive correlation with the biological activity.

### Virtually designed derivatives of Capsazepine

**Figure 2:**
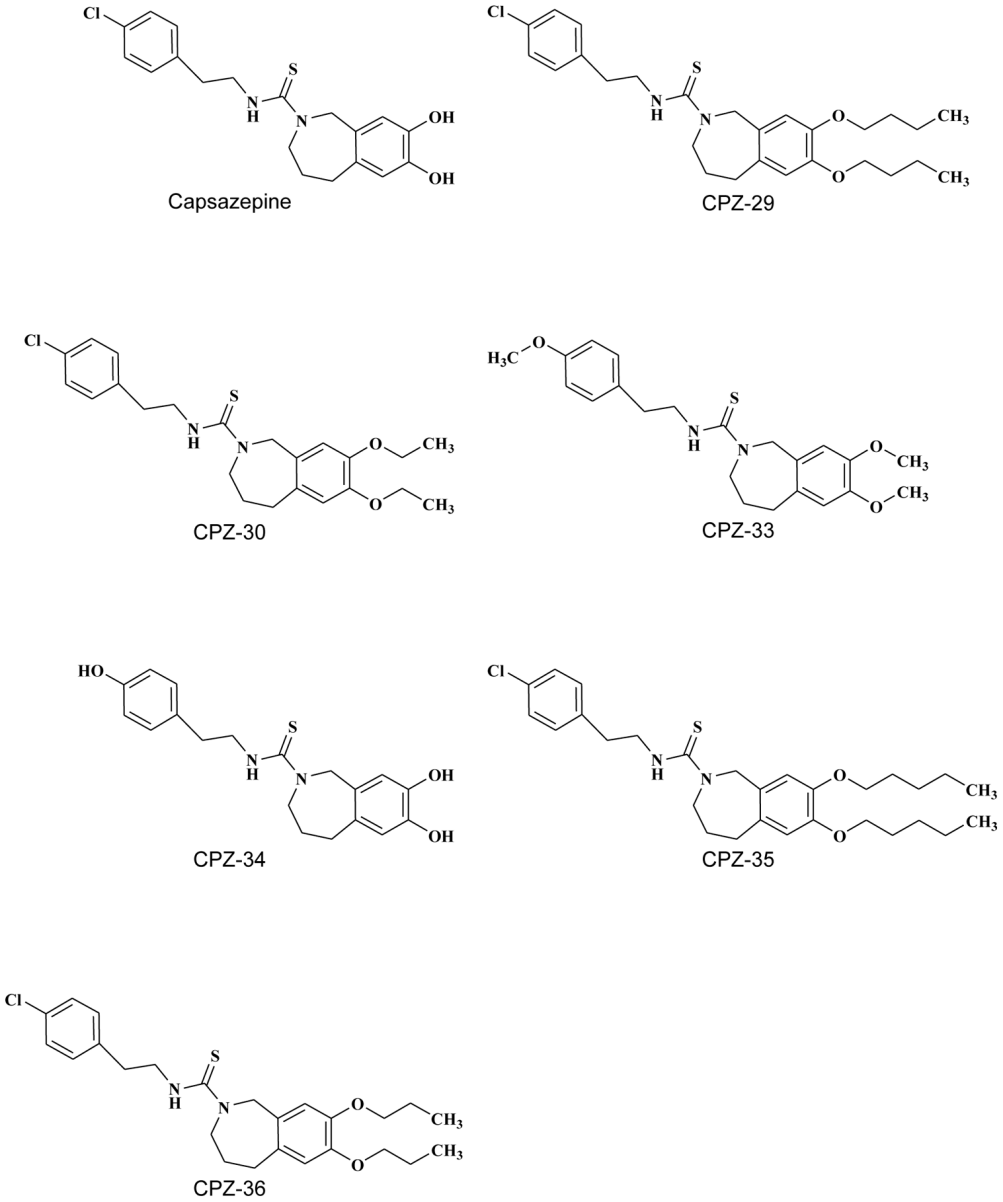
The chemical structure of capsazepine and its active derivatives namely, CPZ-29, CPZ-30, CPZ-33, CPZ-34, CPZ-35 and CPZ-36.

Forty-four capsazepine derivatives were virtually designed and their probable activities were predicted by the developed QSAR model. Virtual screening through derived QSAR model resulted six best hits *e.g*., CPZ-29, CPZ-30, CPZ-33, CPZ-34, CPZ-35 and CPZ-36. Predicted IC_50_ (µM) of capsazpine derivatives are summarized in Table 1 and structure of active capsazepine derivatives are showed in Figure 2.

**Table 1:** Predicted IC_50_ (µM) of capsazepine and its derivatives (query set) calculated from derived QSAR model.

Derivatives	ALogP	Molecular weight	No. of H-bond donor	No. of H-bond acceptor	No. of rotatable bonds	No. of rings	No. of aromatic rings	MF-PSA	Predicted IC_50_ (µM)
CPZ-1	5.945	495.419	1	5	10	3	2	0.217	−216.184
CPZ-2	7.567	551.096	1	5	12	4	3	0.191	−205.677
CPZ-3	6.971	568.041	1	7	11	4	3	0.275	−194.643
CPZ-4	9.077	570.056	2	7	10	4	3	0.277	−123.269
CPZ-5	9.077	570.056	2	7	10	4	3	0.277	−123.269
CPZ-6	5.402	418.937	2	4	7	3	2	0.236	−127.342
CPZ-7	7.076	523.043	1	5	10	4	3	0.204	−106.228
CPZ-8	7.067	481.006	2	4	8	4	3	0.21	−76.0775
CPZ-9	7.05	511.032	2	5	9	4	3	0.215	−48.8401
CPZ-10	6.466	566.111	2	6	13	4	3	0.208	−196.492
CPZ-11	7.485	630.98	2	6	12	4	3	0.202	−192.097
CPZ-12	8.066	621.982	2	6	12	4	3	0.198	−179.463
CPZ-13	7.401	586.529	2	6	12	4	3	0.206	−200.42
CPZ-14	6.631	597.082	2	8	13	4	3	0.282	−193.736
CPZ-15	6.631	597.082	2	8	13	4	3	0.282	−199.539
CPZ-16	6.631	597.082	2	8	13	4	3	0.282	−235.016
CPZ-17	6.737	552.084	2	6	12	4	3	0.215	−206.963
CPZ-18	6.72	582.11	2	7	13	4	3	0.219	−147.44
CPZ-19	5.755	518.068	2	6	13	3	2	0.222	−166.633
CPZ-20	6.211	532.095	2	6	14	3	2	0.216	−166.141
CPZ-21	6.463	546.121	2	6	14	3	2	0.208	−188.019
CPZ-22	7.06	553.069	1	6	11	4	3	0.209	−49.7968
CPZ-23	7.027	613.121	1	8	13	4	3	0.217	−88.9147
CPZ-24	8.405	591.933	1	5	10	4	3	0.186	−145.152
CPZ-25	6.958	503.053	1	5	10	3	2	0.201	−122.442
CPZ-26	10.185	601.239	1	5	19	3	2	0.167	−125.982
CPZ-27	11.553	643.319	1	5	22	3	2	0.154	−100.981
CPZ-28	5.32	440.555	2	5	9	3	2	0.237	−120.574
CPZ-29*	8.501	489.113	1	3	13	3	2	0.134	46.4547
CPZ-30*	6.542	433.007	1	3	9	3	2	0.155	29.7612
CPZ-31	10.326	545.219	1	3	17	3	2	0.119	79.8786
CPZ-32	5.844	404.953	1	3	7	3	2	0.167	−27.6355
CPZ-33*	5.163	400.534*	1	4	8	3	2	0.186	18.2917
CPZ-34*	4.486	358.455*	4	4	5	3	2	0.313	14.1995
CPZ-35*	9.414	517.166*	1	3	15	3	2	0.126	63.1455
CPZ-36*	7.589	461.06*	1	3	11	3	2	0.144	37.6157
CPZ-37	8.036	588.201	2	6	18	3	2	0.192	−133.071
CPZ-38	5.231	504.041	2	6	12	3	2	0.23	−175.905
CPZ-39	5.609	518.068	2	6	12	3	2	0.222	−204.656
CPZ-40	7.123	560.148	2	6	16	3	2	0.203	−149.644
CPZ-41	7.58	574.174	2	6	17	3	2	0.197	−141.379
CPZ-43	8.948	616.254	2	6	20	3	2	0.182	−116.456
CPZ-44	4.515	484.565	1	7	11	3	2	0.267	−77.2436
Capsazepine (*In vitro* activity 26.7982 µM)	5.393	376.9	3	3	5	3	2	0.247	25.631

**Footnote**: *marked compounds indicate predicted active capsazepine derivatives.

Predicted activity of capsazepine was validated by experimental *in vitro* activity.

### Ethics Statement

Primary macrophage cells were isolated from the peritoneal cavities of the healthy female Swiss albino mice as per the approved protocol (AH-2013-06) by the Institutional Animal Ethics Committee (IAEC) of Central Institute of Medicinal and Aromatic Plants, Lucknow followed by the Committee for the Purpose of Control and Supervision of Experimental Animals (CPCSEA), New Delhi, Government of India (Registration No: 400/01/AB/CPCSEA).

### Primary cell culture and treatment

Primary cell culture was carried out as described previously [25]. In brief, the macrophage cells were collected from the peritoneal cavities of mice (8-week-old female Swiss albino mice) after an intra-peritoneal (i.p.) injection of 1.0 mL of 1% peptone (BD Biosciences, USA) 3 days before harvesting. Mice were euthanized by cervical dislocation under ether anesthesia and peritoneal macrophages were obtained by intra-peritoneal injection of Phosphate Buffer Saline (PBS), pH-7.4. Membrane debris was removed by filtering the cell suspensions through sterile gauze. The viability of the cells was determined by trypan blue exclusion and the viable macrophage cells at the concentration of 0.5×106 live cells/mL were used for the experimentation. The cells were suspended in RPMI 1640 medium (Sigma–Aldrich, USA) containing 10% heat-inactivated fetal calf serum (Gibco, USA), 100 µg/mL of penicillin and 100 µg/mL of streptomycin and incubated in a culture plate (Nunc, Germany) at 37°C in 5% CO_2_ in an incubator. Non-adherent cells were removed after 4 h by removing the culture media and the adherent cells were re-suspended in RPMI 1640 medium containing 10% heat-inactivated fetal calf serum. Cells were pretreated with 1, 2.5, 5 and 10 µg/mL of test compounds and standard anti-inflammatory drug, Dexamethasone (Sigma Aldrich, USA) at 5 µg/mL for 30 min. The cells were stimulated with lipopolysaccharide (LPS, 0.5 µg/mL). After incubation with LPS for 24 h, supernatants were collected and immediately frozen at −80°C. Harvested supernatants were tested for quantification of pro-inflammatory mediator TNF-α by ELISA method according to the manufacturer's instructions (BD Biosciences, USA).

### Quantification of pro-inflammatory cytokines

**Figure 3:**
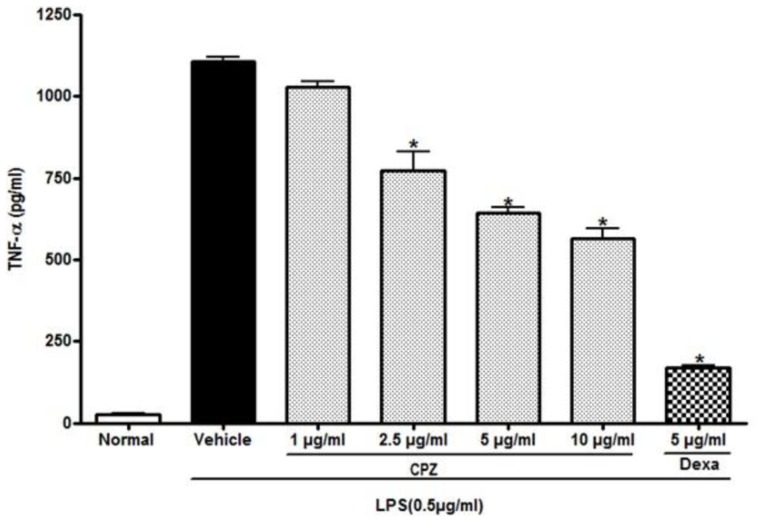
Effect of capsazepine on production of TNF-α in LPS-induced inflammation in macrophage cells (n = 5; p<0.05; * Vehicle versus Treatment).

Quantification of TNF-α at protein level in cell culture supernatant was carried out using Enzyme Immuno Assay (EIA) kits from BD Biosciences, USA following the manufacturer's protocol. Briefly, the ELISA plates (96 well) were coated (100 µL per well) with specific mouse TNF-α capture antibody respectively and incubate overnight at 4°C. The plate was blocked with 200 µL/well assay diluents. Cell Culture supernatant and standard (100 µL) were added into the appropriate coated wells and incubated for 2 h at room temperature (20−25°C). After incubation, the plates were washed thoroughly 5 times with wash buffer. 100 µL of detecting solution (detection antibody and streptavidin HRP) was added in to each well. Seal plate and incubate for 1 h at RT and then the plates were washed thoroughly 5 times with wash buffer. 100 µL of tetramethyl benzidine (TMB) substrate solution to each well and incubate plate (without plate sealer) for 30 min at room temperature in the dark. Add 50 µL of stop solution (2N H_2_SO_4_) to each well. The color density was measured at 450 and 570 nm using a microplate reader (Molecular Devices, USA). Subtract absorbance at 570 nm from absorbance 450 nm. The values of TNF-α were expressed as µg/mL and the IC_50_ values was calculated from vector defined by percentage inhibition values obtained against concentration gradient ranging from 1-10 µg/mL Representative results are depicted in Figure 3 and Table 2.

**Table 2:** Experimental *in vitro* activity for capsazepine against TNF-α.

Compound	LPS (0.5 µg/mL)	Concentration (µg/mL)	TNF-α (pg/mL) Mean±SEM	Percentage Inhibition	IC_50_ (µM)
Normal	-	-	24.60±4.62	-	
Vehicle	√	-	1107.33±14.18	0%	
Capsazepine	√	1 µg	1028.47±18.81	7.1%	26.7982**
	√	2.5 µg	774.67±58.06*	30%	
	√	5 µg	642.47±18.56*	41.9%	
	√	10 µg	566.40±30.83*	48.9%	
Dexamethasone	√	5 µg	167.87±8.42*	84.84%	

**Note**: *indicate significant effect,

**IC_50_ values was calculated from vector defined by percentage inhibition values obtained against concentration gradient ranging from 1-10 µg/mL.

### In vitro cell cytotoxicity

**Figure 4:**
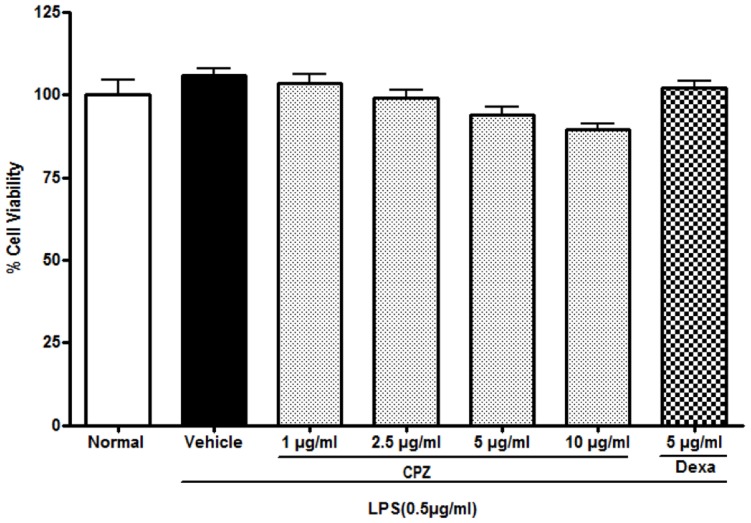
Effect of capsazepine on percent (%) cell viability of macrophage cells using MTT assay.

Cytotoxicity was carried out in macrophage cells using 3-(4,5-dimethylthiazol-2-yl)-2,5-diphenyltetrazolium (MTT) assay. Peritoneal macrophage cells (0.5×10^6^ cell/well) isolated from mice were suspended in RPMI 1640 medium (Sigma, USA) containing 10% heat-inactivated fetal bovine serum (Gibco, USA) and incubated in a culture 96 well plate at 37°C in 5% CO_2_ in an incubator and left overnight to attach. Cells treated with 1% DMSO served as a vehicle control for cell cytotoxicity study. Extracts and capsazepine were dissolved in DMSO. Cells were treated (1, 2.5, 5, 10 µg/mL) and incubated for 24 h at 37°C in 5% CO_2_. After incubation cells with treatment, 20 µl aliquots of MTT solution (5 mg/mL in PBS) were added to each well and left for 4 h. Then, the MTT containing medium carefully removed and the cells were solubilised in DMSO (100 µL) for 10 min. The culture plate was placed on a micro-plate reader (Spectromax plus 384 with Softmax pro v5.3 software; Molecular device, USA) and the absorbance was measured at 550 nm. The amount of color produced is directly proportional to the number of viable cells. Cell cytotoxicity was calculated as the percentage of MTT absorption as follows: Percentage (%) of survival  =  (mean experimental absorbance/mean control absorbance ×100) [26]. The MTT assay results are summarized in Figure 4 and Table 3.

**Table 3:** MTT assay for capsazepine toxicity evaluation.

Compound	LPS (0.5 µg/mL)	Concentration (µg/mL)	% Live Cells Mean±SEM
Normal	-	-	100.00±4.53
Vehicle	√	-	105.89±2.20
Capsazepine	√	1	103.53±2.92
	√	2.5	99.07±2.32
	√	5	93.87±2.46
	√	10	89.47±1.92
Dexamethasone	√	5	102.08±2.02

**Note**: Significant (p<0.05) changes in % live cells were not observed in any treatment.

### Statistical analysis

Results were presented as the means ±SEM and analyzed using GraphPad Prism 4. The ANOVA followed by turkeys multiple comparison tests was used to assess the statistical significance of vehicle verses treatment groups. Results are presented as the means ±SEM. Differences with a p value <0.05 were considered significant. IC_50_ values were calculated from vector defined by percentage inhibition values obtained against a concentration gradient ranging from 1−10 µg/mL.

### Molecular docking

The docking study of selected target and ligands was done by using Autodock Vina v0.8 (Molecular Graphics Lab at The Scripps Research Institute, La Jolla, CA 92037, USA). The 3D crystallographic structure of anti-inflammatory protein target tumor necrosis factor-α (TNF-α) was retrieved through Brookhaven Protein DataBank (PDB) (http://www.pdb.org) (PDB ID: 2AZ5). The crystallographic protein structure of TNF-α complexes with known inhibitor was selected for docking procedure validation by re-docking approach and also to know the standard docking energy and binding site. The valency and hydrogen bonds of the ligands as well as target protein was subsequently satisfied. An extended PDB format, termed as PDBQT file was used for coordinate files that includes atomic partial charges. The software automatically convert PDB file into PDBQT that was further used for docking [27]. Polar hydrogen atoms were added to the protein target to achieve the correct ionisation and tautomeric states of amino acid residues such as HIS, ASP, SER and GLU. For software standardization, native ligand of co-crystallised complex was first extracted and re-docked to its corresponding binding site using AutoDock Vina v0.8. The docking of ligand with receptor TNF-α was performed for three times and the average of consecutive results was taken as final binding/docking energy. Binding pose with the lowest docked energy belongs to the top-ranked cluster was selected as the final model for post-docking analysis with UCSF Chimera v1.5.3 (NCRR, University of California, San Francisco, supported by NIH P41 RR001081) and PyMOL (The PyMOL Molecular Graphics System, Version 1.5.0.4 Schrödinger, LLC, USA).

### Screening through pharmacokinetic properties

Most of drugs in development failed during clinical trials due to poor pharmacokinetics parameters [28], [29]. These properties such as absorption, distribution, metabolism, excretion and toxicity (ADMET) are important in order to determine the success of the compound for human therapeutic use. Some important chemical descriptors correlate well with ADMET properties such as polar surface area (PSA) as a primary determinant of fraction absorption, low molecular weight (MW) for oral absorption. The distribution of the compound in the human body depends on factors such as blood–brain barrier (Log BB), permeability such as apparent Caco-2 permeability, apparent MDCK cell permeability, Log Kp for skin permeability, volume of distribution and plasma protein binding (Log Khsa for Serum protein binding). It has been reported that excretion process that eliminates the compound from human body depends on the molecular weight and octanol–water partition coefficient (LogP). Similarly, rapid renal clearance is associated with small and hydrophilic compounds. The metabolism of most drugs that takes place in the liver is associated with large and hydrophobic compounds. Higher lipophilicity of compounds leads to increased metabolism and poor absorption, along with an increased probability of binding to undesired hydrophobic macromolecules, thereby increasing the potential for toxicity. In spite of some observed exceptions to Lipinski's rule, the property values of the vast majority (90%) of the orally active compounds are within their cut-off limits [30]–[31]. In addition, the bioavailability of derivatives was assessed through topological polar surface area analysis. We calculated the polar surface area (PSA) by using method based on the summation of tabulated surface contributions of polar fragments termed as topological PSA (TPSA). Generally, it has been seen that passively absorbed compounds with a PSA>140 Å^2^ are thought to have low oral bioavailability. Calculations of other important ADME properties of capsazepine derivatives were performed through Discovery Studio v3.5, USA (2013). We also screened capsazepine and its derivatives through TOPKAT toxicity estimation using Discovery Studio v3.5. TOPKAT computes a probable value of toxicity for a submitted chemical structure from a quantitative structure-toxicity relationship (QSTR) equation. The product of a structure descriptors and its corresponding coefficient is the descriptors contribution to the probable toxicity.

## Results and Discussion

In the present work, derivatives of capsazepine were evaluated for their anti-inflammatory activity through the developed QSAR model and docking studies. Structure activity relationship has been denoted by QSAR model showing significant internal prediction (r^2^ = 0.8779) and external prediction (r^2^_pred_ = 0.5865) (Figure 1). A total of 124 known inhibitors of TNF-α were used for QSAR modeling against 57 2D and 3D chemical descriptors. Eight descriptors were found to be significantly responsible for anti-inflammatory activity (Table 1). A forward feed multiple linear regression QSAR model was developed. Both internal and external validations were performed for the developed model. A low residual value for each compound in the dataset defines the degree of correlation between observed and predicted values and the models predictive ability. Screening of derivatives through developed QSAR model indicated that derivatives CPZ-29, CPZ-30, CPZ-33, CPZ-34, CPZ-35 and CPZ-36 showed significant activity in compared to capsazepine's *in vitro* IC_50_ against TNF-α (Table 2). In 1989, it was found that capsazepine and some of its derivatives possessed extraordinary anti-inflammatory activity against COX-2 and inducible nitric oxide (iNO) [11], [12]. In the present work, we report anti-inflammatory activity of 44 virtually designed capsazepine derivatives with lactone ring pharmacophore against pro-inflammatory target TNF-α. The activity of newly designed derivatives were predicted through the developed QSAR model and the derivatives CPZ-33 and CPZ-34 found to be better in activity as compared to capsazepine, whereas CPZ-30 was found close to capsazepine. All six derivatives and parent compound (Figure 2) were further selected for *in silico* target-receptor interaction and ADMET studies.

### Binding affinity study through docking against TNF-α

**Figure 5:**
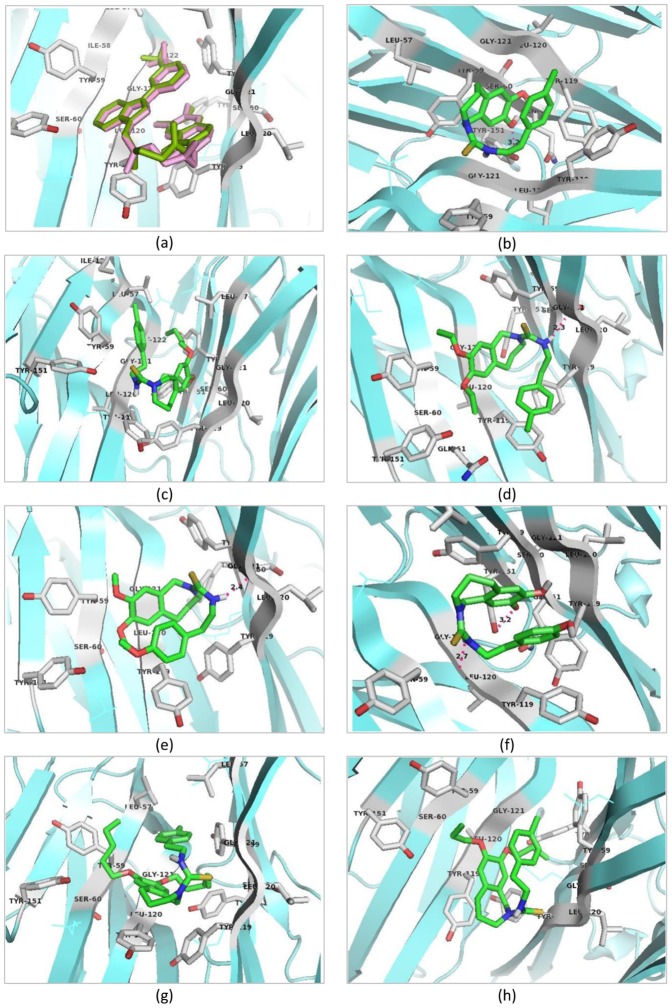
Docking pose of capsazepine and its active derivatives on anti-inflammatory receptor TNF-α (PDB: 2AZ5). (a) Docking protocol standardization by re-docking of co-crystallized ligand on TNF-α with docking energy −9.2 kcal/mol, (b) Capsazepine docked on TNF-α with binding energy −7.3 kcal/mol, (c) CPZ-29 docked on TNF-α with binding energy −7.2 kcal/mol, (d) CPZ-30 docked on TNF-α with binding energy −7.9 kcal/mol, (e) CPZ-33 docked on TNF-α with binding energy −6.8 kcal/mol, (f) CPZ-34 docked on TNF-α with binding energy −7.8 kcal/mol, (g) CPZ-35 docked on TNF-α with binding energy −7.3 kcal/mol and (h) CPZ-36 docked on TNF-α with binding energy −6.9 kcal/mol.

The aim of the molecular docking study was to elucidate whether capsazepine derivatives modulate the anti-inflammatory target and also to identify the binding site against well-known human anti-inflammatory molecular target TNF-α. The native ligand re-docking study indicates that the software predict the reliable results. Thereafter the predicted active derivatives were subjected to molecular docking studies. The docking results provided pertinent information about the binding affinity, binding energy and orientation of ligand-receptor interactions. The docking results are summarized in Table 4. It has been found that capsazepine and its active derivatives bound to the same active site as reported in the PDB protein crystallographic structure database. Capsazepine showed significant binding affinity to TNF-α dimeric structural unit (A and B chain residues) with binding energy of −7.3 kcal/mol, similarly, capsazepine active derivatives CPZ-34, and CPZ-30 showed high binding affinity to TNF-α dimeric structural unit with docking energy of −7.8 and −7.9 kcal/mol, respectively. Docking pose of capsazepine and its active derivatives on receptor TNF-α are showed in Figure 5. On the other hand, capsazepine derivatives CPZ-29 and CPZ-35 showed moderate binding affinity to TNF-α dimeric structural unit with binding energy of −7.2 and −7.3 kcal/mol, respectively. The capsazepine derivatives CPZ-33 and CPZ-36 showed low binding affinity to TNF-α dimeric structural unit with binding energy of −6.8 and −6.9 kcal/mol, respectively. The chemical nature of capsazepine & its derivatives binding site amino acid residues on TNF-α dimeric structural unit (Chain A & B) were aliphatic (*e.g*., LEU-57, LEU-120, GLY-121, GLY-122), hydroxyl group containing (*e.g*., SER-60), and aromatic (*e.g.*, TYR-59 and TYR-119). The capsazepine, CPZ-30, CPZ-33 and CPZ-34 showed H-bonds with the TNF-α dimeric unit residues, this suggest high structural stability and may lead to high inhibitory activity of capsazepine and its derivatives on TNF-α active site.

**Table 4:** Comparison of binding affinity of capsazepine and its active derivatives in terms of docking energy and binding site residues against anti-inflammatory receptor TNF-α (PDB: 2AZ5).

Compounds	Docking binding energy (kcal/mol)	Chain ID (dimer form)	Binding pocket residues within 4 Å radius	Interacting residues and length (4 Å)
Re-docking of bound inhibitor-307* in TNF-α dimer complex form	−9.1	Chain A:	LEU-57, TYR-59, SER-60, TYR-119, LEU-120, GLY-121, GLY-122, TYR-151	
		Chain B:	LEU-57, TYR-59, TYR-119, LEU-120, GLY-121	
Capsazepine	−7.3	Chain A:	LEU-59, TYR-119, LEU-120, GLY-121	
		Chain B:	LEU-57, TYR-59, SER-60, GLN-61, TYR-119, LEU-120, GLY-121, TYR-151	Chain B: LEU-120 (2.3)
CPZ-29	−7.2	Chain A:	LEU-57, TYR-59, TYR-119, LEU-120, GLY-121, GLY-122, TYR-151, ILE-155	
		Chain B:	LEU-57, TYR-59, SER-60, TYR-119, LEU-120, GLY-121, TYR-151	
CPZ-30	−7.9	Chain A:	TYR-59, SER-60, GLN-61, TYR-119, LEU-120, GLY-121, TYR-151	
		Chain B:	TYR-59, SER-60, TYR-119, LEU-120, GLY-121, TYR-151	Chain B: LEU-120 (2.3)
CPZ-33	−6.8	Chain A:	TYR-59, SER-60, TYR-119, LEU-120, GLY-121, TYR-151	
		Chain B:	TYR-59, SER-60, TYR-119, LEU-120, GLY-121	Chain B: LEU-120 (2.7)
CPZ-34	−7.8	Chain A:	TYR-59, TYR-119, LEU-120, GLY-121	Chain A: LEU-120 (2.7) and
		Chain B:	LEU-57, TYR-59, SER-60, GLN-61, TYR-119, LEU-120, GLY-121, TYR-151	Chain B: TYR-151 (3.2)
CPZ-35	−7.3	Chain A:	LEU-57, TYR-59, SER-60, TYR-119, LEU-120, GLY-121TYR-151	
		Chain B:	LEU-57, TYR-59, SER-60, TYR-119, LEU-120, GLY-121, TYR-151	
CPZ-36	−6.9	Chain A:	TYR-59, SER-60, TYR-119, LEU-120, GLY-121, TYR-151	
		Chain B:	TYR-59, SER-60, TYR-119, LEU-120, GLY-121, TYR-151	

**Note**: “-” represent no H-bond and * refer TNF-α dimer inhibitor-307 name (6,7-DIMETHYL-3-[(METHYL{2-[METHYL({1-[3-(TRIFLUOROMETHYL)PHENYL]- 1H-INDOL-3-YL}METHYL)AMINO]ETHYL}AMINO)METHYL]- 4H-CHROMEN-4-ONE).

### Bioavailability and ADME parameters screening for drug likeness

**Figure 6:**
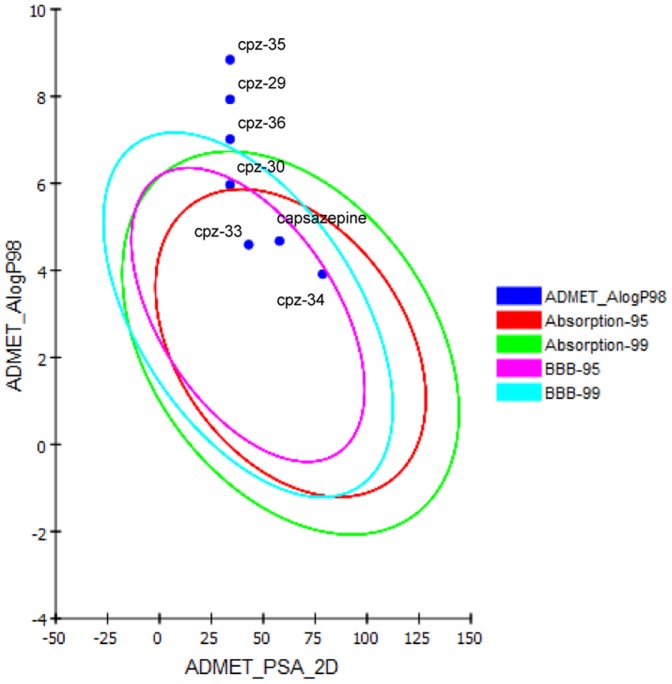
Plot of polar surface area (PSA) versus ALogP for capsazepine and its derivatives showing the 95% and 99% confidence limit ellipses corresponding to the blood brain barrier (BBB) and intestinal absorption.

The compound's good absorption or permeation through blood brain barrier is measure by its LogP that must be less than 5 [22]–[23]. Results of pharmacokinetic screening revealed that capsazepine and its most active derivatives CPZ-33 and CPZ-34 followed the Lipinki's rule of five for oral bioavailability. However CPZ-29, CPZ-30, and CPZ-36 showed lipophilic nature due to high LogP value, while compound CPZ-35 showed both high lipophilicity and low membrane permeability due to high LogP and molecular weight. These ADMET screening results are summarized in Table 5 and 6. The ADME descriptors of capsazepine and its derivatives were calculated for drug likeness studies. The intestinal absorption and blood brain barrier penetration were predicted by developing an ADME model using descriptors 2D PSA and AlogP98 that include 95% and 99% confidence ellipses. These ellipses define regions where well-absorbed compounds are expected to be found. The results of DS-ADME model screening showed that capsazepine derivatives CPZ-33 and CPZ-34 possess 99% confidence levels for human intestinal absorption and blood brain barrier (BBB) penetration. Similarly, another predicted active capsazepine derivative CPZ-30 also showed 99% confidence level for intestinal absorption and 95% confidence level for BBB penetration. The capsazepine derivatives CPZ-29, CPZ-35 and CPZ-36 fall outside the ADME model ellipses filter, which indicate its poor intestinal absorption and BBB penetration ability. The plot of polar surface area and ALogP fpr capsazepine and its derivatives are represented in Figure 6.

**Table 5:** Compliance of capsazepine and its derivatives to the computational parameters of oral bioavailability (Lipinski's rule of five).

Compound	Oral bioavailability: TPSA (<140 Å^2^)	MW (<500)	ALog P (≤5)	H-bond donor (≤5)	H-bond acceptor (≤10)	Rule of 5 violations
Capsazepine	87.82	376.909	5.393	3	3	0
CPZ-29	74.47	489.113	8.501	1	3	1
CPZ-30	74.47	433.007	6.542	1	3	1
CPZ-33	83.7	400.534	5.163	1	4	0
CPZ-34	116.7	358.455	4.486	4	4	0
CPZ-35	74.47	517.166	9.414	1	3	2
CPZ-36	74.47	461.06	7.589	1	3	1

**Footnote**: A, absorption; D, distribution; M, metabolism; E, excretion; TPSA, topological polar surface area; MW, molecular weight; LogP  =  octanol/water partition coefficient, a measure for lipophilicity.

**Table 6:** Compliance of capsazepine and its active derivatives to the computational parameters of pharmacokinetics (ADME).

Compound	AlogP	PSA_2D	Plasma protein binding	Hepatotoxicity	CYP2D6 binding	Aqueous solubility	BBB penetration	Intestinal absorption
Capsazepine	4.674	57.793	True (highly bounded)	True (toxic)	False (non-inhibitor)	2 (low)	1(good)	0 (good)
CPZ-29	7.929	34.022	True (highly bounded)	False (non-toxic)	False (non-inhibitor)	1 (poor)	4 (undefined)	3 (very poor)
CPZ-30	5.969	34.022	True (highly bounded)	False (non-toxic)	False (non-inhibitor)	1 (poor)	0 (very good)	1 (moderate)
CPZ-33	4.591	42.952	True (highly bounded)	True (toxic)	False (non-inhibitor)	2 (low)	1 (good)	0 (good)
CPZ-34	3.914	78.609	False (poorly bounded)	False (non-toxic)	False (non-inhibitor)	3 (good)	2 (medium)	0 (good)
CPZ-35	8.841	34.022	True (highly bounded)	False (non-toxic)	False (non-inhibitor)	0 (very poor)	4 (undefined)	3 (very poor
CPZ-36	7.016	34.022	True (highly bounded)	False (non-toxic)	False (non-inhibitor)	1(poor)	4 (undefined)	3 (very poor

**Abbreviations**: AlogP, the logarithm of the partition coefficient between n-octanol and water; PSA, polar surface area, CYP450 cytochrome P450, PPB plasma protein binding, BBB blood brain barrier.

### Toxicity risks assessment

The USFDA (US FDA, United States Food and Drug Administration) standard toxicity risk predictor software TOPKAT (Discovery Studio, Accelrys, USA) locates fragments within the compound that indicate a potential threat to toxicity risk [26]. Toxicity screening results of TOPKAT for capsazepine and its derivatives showed that studied compounds possess no risk of carcinogenicity, mutagenicity and skin irritation, however it possess high developmental or reproductive toxicity potential at high doses or long term therapeutic use in human. The capsazepine and its derivatives CPZ-30, CPZ-33 and CPZ-34 showed strong skin sensitization capacity. Similarly, capsazepine derivatives CPZ-33 and CPZ-34 also showed mild ocular irritancy. Other detail predicted toxicity parameters are summarized in Table 6. The results of toxicity risk for capsazepine and its active derivatives showed moderate to good drug score, in compared with capsazepine (Table 7a). Similarly, toxicity screening results of USFDA rodent carcinogenicity, Ames mutagenicity, developmental toxicity potential, aerobic biodegradability, ocular irritancy and skin irritancy also showed positive response to capsazepine and its derivatives (Table 7b).

**Table 7:** A) Compliance of capsazepine and its derivatives to the computational parameters of toxicity risk; B) Compliance of capsazepine and its derivatives to the computational parameters of USFDA rodent carcinogenicity, Ames mutagenicity, developmental toxicity potential, aerobic biodegradability, ocular irritancy and skin irritancy.

A							
Parameters	Capsazepine	CPZ-29	CPZ-30	CPZ-33	CPZ-34	CPZ-35	CPZ-36
Rate oral LD_50 (g/kg body weight)_	0.266693	0.629811	0.466001	0.729137	0.520863	0.639541	0.437826
Rat inhalational LC_50__(mg/m3/h)_	2.02828	8.37927	3.77263	2.19292	0.680172	7.31381	10.4836
Daphnia EC_50__(mg/L)_	0.197718	0.0134987	0.039166	0.0437572	0.442085	0.00403665	0.0538994
Rat chronic LOAEL _(g/kg body weight)_	0.0276461	0.0416238	0.0373369	0.0253561	0.138026	0.0548522	0.039561
Fathead minnow LC_50__(g/L)_	0.000319894	2.3249e−06	6.13319e−05	0.000164794	0.0025682	4.86682e−07	1.10824e−05
**Carcinogenic potency TD_50__(mg/kg body weight/day)_**							
Mouse	29.6009	43.3234	18.3346	13.7675	27.3028	46.5307	27.9847
Rat	19.0951	11.3013	7.5627	4.52229	30.9968	30.9247	7.17525
Rat maximum tolerated dose _(g/kg body weight)_	0.77649	0.225911	0.175138	0.0811242	1.37787	0.252173	0.201686

B							
Parameters	Capsazepine	CPZ-29	CPZ-30	CPZ-33	CPZ-34	CPZ-35	CPZ-36
Mouse female	Non-Carcinogen	Non-Carcinogen	Non-Carcinogen	Non-Carcinogen	Non-Carcinogen	Non-Carcinogen	Non-Carcinogen
Mouse male	Carcinogen	Carcinogen	Carcinogen	Non-Carcinogen	Non-Carcinogen	Carcinogen	Carcinogen
Rat female	Non-Carcinogen	Non-Carcinogen	Non-Carcinogen	Non-Carcinogen	Non-Carcinogen	Non-Carcinogen	Non-Carcinogen
Rat male	Non-Carcinogen	Non-Carcinogen	Non-Carcinogen	Non-Carcinogen	Non-Carcinogen	Non-Carcinogen	Non-Carcinogen
Ames mutagenicity	Non-Mutagen	Non-Mutagen	Non-Mutagen	Non-Mutagen	Non-Mutagen	Non-Mutagen	Non-Mutagen
Developmental toxicity potential	Toxic	Toxic	Toxic	Toxic	Toxic	Toxic	Toxic
Aerobic biodegradability	Non-Degradable	Non-Degradable	Non-Degradable	Non-Degradable	Non-Degradable	Non-Degradable	Non-Degradable
Ocular irritancy	Severe	Severe	Severe	Mild	Mild	Severe	Severe
Skin irritancy	None	None	None	None	None	None	None

**Abbreviations**: USFDA, United States Food and Drug Administration.

**Abbreviations**: EC_50_, effective concentration 50%; LC_50_, lethal concentration 50%; LD_50_, lethal dose 50%; LOAEL, lowest observed adverse effect level; TD_50_, tumorigenic dose 50%.

### Anti-inflammatory potential of Capsazepine

To examine the effects of capsazepine on LPS-induced pro-inflammatory cytokine TNF-α in macrophages, culture supernatant from various treatment groups were used to determine their production. Treatment of capsazepine inhibited (p<0.05) the production of LPS-induced inflammatory mediator in dose dependant manner. (Figure 3, Table 2).

### Measurement of the cell viability

The *in vitro* effect of capsazepine on cell viability in peritoneal macrophage cells isolated from mice was evaluated using MTT assay. The significant change in percent live cell population was not observed (p<0.05) at any concentration of the treatment when compared with normal cells (Figure 4, Table 3).

## Conclusions

The experimental *in vitro* evaluation of capsazepine against pro-inflammatory mediator TNF-α indicated that capsazepine mediate significant inhibitory effect on the TNF-α. The predicted activity of capsazepine was comparable with the experimental results. Ligand-based virtual screening through developed QSAR model resulted in six best hits for capsazepine derivatives CPZ-29, CPZ-30, CPZ-33, CPZ-34, CPZ-35 and CPZ-36. The capsazepine derivatives CPZ-33 and CPZ-34 showed good predicted activity and binding affinity to TNF-α in compared with capsazepine. Docking results indicate that the major influencing factors of molecular interactions between TNF-α and capsazepine and its derivatives were H-bonds, hydrophobic and electrostatic interactions. Results of oral bioavailability (rule of five), ADME and toxicity risk profiling were within the acceptable limit for capsazepine derivatives CPZ-33 and CPZ-34. These compounds as such and on further lead optimization may guide to designing of novel TNF-α inhibitors.

## Supporting Information

File S1 Contains Table S1, Structure, experimental IC_50_ (µM), predicted IC_50_ (µM) and residual of training set compounds. Table S2, Structure, experimental IC_50_ and predicted IC_50_ of test set compounds. Table S3, Details of derived QSAR model equation based on multiple linear regression.
(DOC)
