# Supplementary material for: QSAR and Docking Studies on Capsazepine Derivatives for Immunomodulatory and Anti-Inflammatory Activity
Source: PLoS One. 2014 Jul 8;9(7):e100797. doi: 10.1371/journal.pone.0100797 (PMC4086833; doi:10.1371/journal.pone.0100797)
Supplement: File S1 — Contains Table S1, Structure, experimental IC50 (µM), predicted IC50 (µM) and residual of training set compounds. Table S2, Structure, experimental IC50 and predicted IC50 of test set compounds. Table S3, Details of derived QSAR model equation based on multiple linear regression. (DOC) [file pone.0100797.s001.doc]

Table S1: Structure, experimental IC_50_ (µM), predicted IC_50_ (µM) and residual of training set compounds.

| **Compound** | **Structure** | **Exp.IC_50_ (µM)** | **Pre. IC_50_ (µM)** | **Residual** | **Reference no.** |
| --- | --- | --- | --- | --- | --- |
| 63 |  | 0.09 | 0.498392 | -0.408392 | [22] |
| 12a |  | 0.1 | 2.12594 | -2.02594 | [21] |
| 62 |  | 0.12 | 0.12 | 2.30425e-08 | [22] |
| 10 |  | 0.32 | 1.55286 | -1.23286 | [22] |
| 56 |  | 0.4 | 4.52938 | -4.12937 | [22] |
| 67 |  | 0.45 | 1.68286 | -1.23286 | [22] |
| 11 |  | 0.6 | 6.94853 | -6.34853 | [21] |
| 13 |  | 0.6 | 0.6 | -3.28912e-08 | [21] |
| 8a |  | 0.6 | -2.62982 | 3.22982 | [21] |
| 39 |  | 0.78 | 14.5982 | -13.8182 | [22] |
| 3bb |  | 0.9 | 3.36572 | -2.46572 | [20] |
| 5b |  | 0.9 | 10.4492 | -9.54916 | [19] |
| 14 |  | 1 | 7.48301 | -6.48301 | [20] |
| 35 |  | 1.1 | 9.2459 | -8.1459 | [22] |
| 23 |  | 1.3 | 2.41315 | -1.11315 | [22] |
| 33 |  | 1.7 | -1.46993 | 3.16993 | [22] |
| 10b |  | 1.7 | 2.40422 | -0.704218 | [23] |
| 9a |  | 2 | 10.4504 | -8.45043 | [21] |
| 41 |  | 2.1 | -0.500322 | 2.60032 | [22] |
| 3nn |  | 2.2 | -1.38377 | 3.58377 | [20] |
| 5a |  | 2.6 | 5.88146 | -3.28146 | [19] |
| 22 |  | 2.7 | -3.38911 | 6.08911 | [22] |
| 3z |  | 2.9 | 7.11429 | -4.21429 | [19] |
| 3m |  | 2.9 | -1.49019 | 4.39019 | [19] |
| 3bb |  | 3 | 0.808596 | 2.1914 | [19] |
| 44 |  | 3 | 5.55682 | -2.55682 | [24] |
| 3k |  | 3 | -1.78567 | 4.78567 | [19] |
| 28 |  | 3.2 | 3.2 | -4.85691e-09 | [22] |
| 3j |  | 3.4 | 2.45718 | 0.942821 | [19] |
| 3y |  | 3.9 | -0.314291 | 4.21429 | [19] |
| 5d |  | 3.9 | -0.776694 | 4.67669 | [19] |
| 10a |  | 4 | 10.05 | -6.05 | [19] |
| 78 |  | 5 | 7.54496 | -2.54496 | [24] |
| 4 |  | 5 | 5 | 1.77531e-09 | [24] |
| 15 |  | 5 | 3.87575 | 1.12425 | [24] |
| 3ll |  | 6 | 12.6535 | -6.65345 | [20] |
| 3e |  | 6 | 7.83687 | -1.83687 | [19] |
| 45 |  | 6 | 13.8221 | -7.82208 | [24] |
| 6b |  | 6.5 | 5.26714 | 1.23286 | [19] |
| 3j |  | 7.1 | 12.076 | -4.97596 | [20] |
| 16 |  | 7.2 | 5.96714 | 1.23286 | [22] |
| 3u |  | 7.6 | 11.5705 | -3.97046 | [19] |
| 3r |  | 7.9 | 6.50477 | 1.39523 | [20] |
| 3g |  | 8.1 | 2.04709 | 6.05291 | [19] |
| 12 |  | 9 | 9.70422 | -0.704218 | [24] |
| 43 |  | 9 | 5.10412 | 3.89588 | [24] |
| 76 |  | 9 | 7.54496 | 1.45504 | [24] |
| 4a |  | 9 | 5.13498 | 3.86502 | [20] |
| 10c |  | 10 | 12.3151 | -2.31513 | [19] |
| 3c |  | 10 | 13.2502 | -3.25017 | [19] |
| 16h |  | 10 | 18.4504 | -8.45043 | []23] |
| 16f |  | 10 | 5.82132 | 4.17868 | [23] |
| 77 |  | 10 | 9.83265 | 0.167347 | [24] |
| 3u |  | 10 | 9.93068 | 0.0693197 | [20] |
| 71 |  | 10.3 | 4.52938 | 5.77063 | [22] |
| 11 |  | 11 | 10.2958 | 0.704218 | [24] |
| 3p |  | 11 | 21.1693 | -10.1693 | [20] |
| 9b |  | 12 | 10.1646 | 1.83538 | [21] |
| 3q |  | 12 | 13.3636 | -1.36365 | [19] |
| 4b |  | 12 | 17.0979 | -5.09788 | [20] |
| 3dd |  | 12 | 17.7085 | -5.7085 | [20] |
| 3q |  | 13 | 16.4922 | -3.4922 | [20] |
| 3x |  | 13 | 11.2761 | 1.72392 | [20] |
| 16 |  | 13 | 12.8655 | 0.134475 | [24] |
| 9c |  | 13 | 10.6567 | 2.34331 | [21] |
| 3dd |  | 14 | 15.741 | -1.74104 | [19] |
| 16b |  | 14 | 16.6022 | -2.6022 | [23] |
| 35 |  | 14 | 14.6551 | -0.655077 | [24] |
| 3g |  | 15 | 14.8412 | 0.158815 | [20] |
| 17 |  | 15 | 11.9665 | 3.03349 | [24] |
| 49 |  | 15 | 16.8964 | -1.89641 | [24] |
| 17a |  | 15 | 13.9241 | 1.07595 | [23] |
| 15c |  | 15 | 18.6516 | -3.65162 | [23] |
| 53 |  | 16.5 | 17.2042 | -0.704218 | [22] |
| 3ff |  | 17 | 11.9359 | 5.06411 | [20] |
| 17b |  | 17 | 19.7172 | -2.7172 | [23] |
| 8c |  | 17.9 | 20.7566 | -2.85661 | [21] |
| 3t |  | 18 | 25.0023 | -7.00234 | [20] |
| 49 |  | 18.7 | 21.1842 | -2.48419 | [22] |
| 11a |  | 19 | 17.7671 | 1.23286 | [19] |
| 3aa |  | 19 | 13.9256 | 5.07441 | [20] |
| 3v |  | 20 | 18.999 | 1.00096 | [19] |
| 50 |  | 20 | 16.9487 | 3.05133 | [24] |
| 3mm |  | 22 | 18.8215 | 3.17848 | [20] |
| 3kk |  | 22 | 9.73368 | 12.2663 | [19] |
| 12b |  | 23 | 26.6706 | -3.67057 | [19] |
| 72 |  | 25 | 16.0242 | 8.97581 | [24] |
| 74 |  | 25 | 25 | -1.57414e-08 | [24] |
| 73 |  | 25 | 25 | 2.00813e-08 | [24] |
| 71 |  | 25 | 17.0546 | 7.94537 | [24] |
| 37 |  | 25 | 24.9953 | 0.00470182 | [24] |
| 68 |  | 25 | 31.8555 | -6.85546 | [24] |
| 36 |  | 25 | 33.0863 | -8.08628 | [24] |
| 42 |  | 25 | 24.5963 | 0.40369 | [24] |
| 69 |  | 25 | 24.5916 | 0.408392 | [24] |
| 67 |  | 25 | 34.1432 | -9.14315 | [24] |
| 82 |  | 25 | 22.9036 | 2.09641 | [24] |
| 55 |  | 25 | 26.5633 | -1.56331 | [24] |
| 66 |  | 25 | 18.517 | 6.48301 | [24] |
| 3jj |  | 28 | 29.2329 | -1.23286 | [24] |
| 3s |  | 28 | 24.864 | 3.13599 | [20] |
| 3a |  | 28 | 27.2277 | 0.772279 | [20] |
| 52 |  | 28.3 | 29.9443 | -1.6443 | [22] |
| 16e |  | 29 | 22.6312 | 6.36882 | [23] |
| 16d |  | 29 | 27.4738 | 1.52625 | [23] |
| 8a |  | 30 | 30.5644 | -0.564421 | [19] |
| 8e |  | 31.3 | 30.5595 | 0.740463 | [21] |
| 3i |  | 33 | 33.8636 | -0.86355 | [19] |
| 28 |  | 33 | 36.8609 | -3.86091 | [24] |
| 3b |  | 34 | 26.705 | 7.29504 | [20] |
| 27 |  | 35 | 38.1591 | -3.15906 | [24] |
| 18 |  | 35 | 31.5763 | 3.42365 | [24] |
| 3w |  | 36 | 34.1484 | 1.85165 | [19] |
| 9a |  | 36 | 33.721 | 2.279 | [23] |
| 30 |  | 37 | 33.5428 | 3.45722 | [24] |
| 10a |  | 37 | 33.0702 | 3.92981 | [23] |
| 11b |  | 38 | 34.3294 | 3.67057 | [19] |
| 29 |  | 38 | 34.8409 | 3.15906 | [24] |
| 1 |  | 38 | 34.2569 | 3.7431 | [19] |
| 8d |  | 41.6 | 30.0675 | 11.5325 | [21] |
| 46 |  | 41.7 | 32.1844 | 9.5156 | [22] |
| 34 |  | 48 | 42.5926 | 5.40739 | [24] |
| 3cc |  | 48 | 47.3325 | 0.667491 | [19] |
| 55 |  | 48.4 | 41.865 | 6.53498 | [22] |
| 5 |  | 50 | 50 | 4.53134e-08 | [24] |

Table S2: Structure, experimental IC_50_ and predicted IC_50_ of test set compounds.

| **Compound** | **Structure** | **Exp. IC_50_ µM** | **Pre. IC_50_ µM** | **Reference No.** |
| --- | --- | --- | --- | --- |
| 38 |  | 1.7 | 5.07753 | [22] |
| 4b |  | 11.6 | 19.0038 | [21] |
| 3w |  | 20 | 19.6586 | [20] |
| 3f |  | 12 | 5.61845 | [19] |
| 17d |  | 13 | 15.615 | [23] |
| 17c |  | 20 | 32.222 | [23] |
| 3r |  | 21 | 22.6887 | [19] |
| 58 |  | 40.1 | 46.8263 | [22] |
| 21 |  | 20 | 28.1881 | [24] |
| 50 |  | 4.9 | 31.1353 | [22] |
| 3ee |  | 26 | 28.8715 | [19] |
| 3ll |  | 10 | 10.7223 | [19] |
| 3z |  | 12 | 1.50617 | [20] |
| 70 |  | 25 | 19.3423 | [24] |
| 31 |  | 55 | 70.7432 | [24] |
| 3cc |  | 6.2 | 22.013 | [20] |
| 15b |  | 14 | 37.2988 | [23] |
| 57 |  | 4.6 | 15.7818 | [22] |
| 60 |  | 9.8 | 11.5074 | [22] |
| 6d |  | 7.1 | 20.492 | [19] |
| 75 |  | 4 | 9.83265 | [24] |
| 3ii |  | 35 | 21.765 | [19] |

Table S3: Details of derived QSAR model equation based on multiple linear regression.

| Multiple Linear Regression Model **TNF** has been created. It contains the following equation: |
| --- |
| [Predicted IC_50_ (µM) ] = -14.38 - 3.084 * [ **ALogP** ] + 0.06609 * [ **Molecular_Weight** ] - 2.684 * [ **Num_H_Donors** ] - 7.081 * [ **Num_H_Acceptors** ] + 8.984 * [ **Num_RotatableBonds** ] - 27.55 * [ **Num_Rings** ] + 39.81 * [ **Num_AromaticRings** ] + 39.31 * [ **Molecular_FractionalPolarSurfaceArea** ] - 14.41 * [ Count<ECFP_6:642810091> ] + 41.73 * [ Count<ECFP_6:-992506539> ] - 22.31 * [ Count<ECFP_6:1559650422> ] - 7.822 * [ Count<ECFP_6:-1100000244> ] - 29.2 * [ Count<ECFP_6:734603939> ] + 25.88 * [ Count<ECFP_6:-1925046727> ] - 21.51 * [ Count<ECFP_6:-1897341097> ] + 100.5 * [ Count<ECFP_6:670515721> ] - 41.45 * [ Count<ECFP_6:-1074141656> ] - 18.62 * [ Count<ECFP_6:914325265> ] - 97.97 * [ Count<ECFP_6:-762035154> ] + 1.292 * [ Count<ECFP_6:-1793471910> ] + 44.49 * [ Count<ECFP_6:-1658273810> ] + 69.29 * [ Count<ECFP_6:-1672512695> ] - 4.91 * [ Count<ECFP_6:-1789102870> ] - 17.13 * [ Count<ECFP_6:1731843802> ] + 28.79 * [ Count<ECFP_6:863188371> ] + 23.62 * [ Count<ECFP_6:-1236483485> ] - 22.3 * [ Count<ECFP_6:-1571351141> ] - 20.14 * [ Count<ECFP_6:849109985> ] - 40.95 * [ Count<ECFP_6:-182236392> ] - 32.25 * [ Count<ECFP_6:-1072294614> ] - 26.89 * [ Count<ECFP_6:-1059365320> ] - 12.88 * [ Count<ECFP_6:683445015> ] + 0.4063 * [ Count<ECFP_6:-427397688> ] + 13.13 * [ Count<ECFP_6:1996767644> ] + 6.379 * [ Count<ECFP_6:-786013480> ] + 47.31 * [ Count<ECFP_6:-176455838> ] + 14.24 * [ Count<ECFP_6:865379614> ] + 46.72 * [ Count<ECFP_6:-181568884> ] + 25.81 * [ Count<ECFP_6:859796174> ] - 5.802 * [ Count<ECFP_6:1997021792> ] + 13.72 * [ Count<ECFP_6:-1684014506> ] - 72.34 * [ Count<ECFP_6:1311676480> ] + 126 * [ Count<ECFP_6:2106656448> ] - 10.19 * [ Count<ECFP_6:-1006447107> ] + 21.02 * [ Count<ECFP_6:-167460056> ] + 10.78 * [ Count<ECFP_6:-154530762> ] + 9.597 * [ Count<ECFP_6:-949601813> ] + 15.34 * [ Count<ECFP_6:1430791942> ] + 0.954 * [ Count<ECFP_6:-1553424473> ] - 3.973e-002 * [ Count<ECFP_6:-89299614> ] - 28.91 * [ Count<ECFP_6:-161045802> ] + 44.4 * [ Count<ECFP_6:1336666212> ] - 7.481 * [ Count<ECFP_6:864909220> ] - 28.57 * [ Count<ECFP_6:-175882072> ] + 1.02 * [ Count<ECFP_6:1408898974> ] + 8.931 * [ Count<ECFP_6:1760125606> ] + 19.53 * [ Count<ECFP_6:2084468150> ] + 0.5962 * [ Count<ECFP_6:-1910270391> ] + 5.84 * [ Count<ECFP_6:-817402818> ] + 153.1 * [ Count<ECFP_6:1849632304> ] - 60.52 * [ Count<ECFP_6:1337040050> ] - 13.26 * [ Count<ECFP_6:-1166853945> ] + 15.18 * [ Count<ECFP_6:1335691903> ] - 35.48 * [ Count<ECFP_6:1564392544> ] - 4.83 * [ Count<ECFP_6:1770731391> ] + 28.81 * [ Count<ECFP_6:1571214559> ] - 7.776 * [ Count<ECFP_6:-1792133435> ] + 9.325 * [ Count<ECFP_6:1745066357> ] - 2.439 * [ Count<ECFP_6:1945129186> ] - 96.99 * [ Count<ECFP_6:1732465867> ] + 7.098 * [ Count<ECFP_6:737975134> ] - 3.785 * [ Count<ECFP_6:381897404> ] - 13.09 * [ Count<ECFP_6:-234452311> ] + 21.52 * [ Count<ECFP_6:-1795525632> ] - 32.2 * [ Count<ECFP_6:-175021654> ] + 5.672 * [ Count<ECFP_6:-468366781> ] - 16.92 * [ Count<ECFP_6:1224255611> ] - 1.345 * [ Count<ECFP_6:-546338324> ] - 12.28 * [ Count<ECFP_6:1919300267> ] - 39.32 * [ Count<ECFP_6:1169886677> ] + 8.295 * [ Count<ECFP_6:-2024255407> ] - 4.504 * [ Count<ECFP_6:-712883311> ] + 82.21 * [ Count<ECFP_6:1336304100> ] + 1.125 * [ Count<ECFP_6:655739385> ] + 17.04 * [ Count<ECFP_6:-1884411803> ] - 159.8 * [ Count<ECFP_6:440818364> ] + 71.24 * [ Count<ECFP_6:2132365030> ] + 20.12 * [ Count<ECFP_6:-1046436026> ] - 22.14 * [ Count<ECFP_6:-219423964> ] |
|  |
